# Unique Findings of Retrograde Double-Balloon Enteroscopy in a Rare Adult Case of Ileal Duplication

**DOI:** 10.14309/crj.0000000000000511

**Published:** 2020-12-16

**Authors:** Katsuya Endo, Ken Nihei, Keigo Murakami, Kennichi Satoh

**Affiliations:** 1 Division of Gastroenterology, Tohoku Medical and Pharmaceutical University School of Medicine, Sendai, Miyagi, Japan; 2 Division of Pathology, Tohoku Medical and Pharmaceutical University School of Medicine, Sendai, Miyagi, Japan

## CASE REPORT

Gastrointestinal (GI) duplications are congenital malformations that can be present anywhere from the esophagus to the anus. GI duplications are usually observed in pediatric patients, and their diagnosis in adulthood is quite rare. Preoperative diagnosis of GI duplication is difficult, particularly in the small intestine.^1^ We encountered a rare adult case of duplication of the ileum that was accidentally diagnosed in an asymptomatic state using double-balloon endoscopy (DBE).

**Figure 1.**
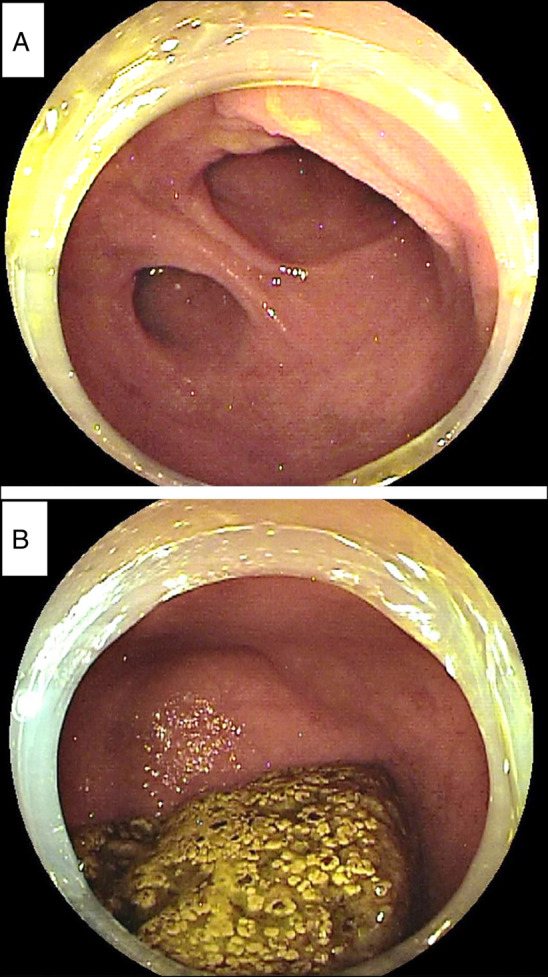
Retrograde double-balloon enteroscopy showing (A) a bifurcated lumen in the ileum approximately 100 cm from the ileocecal valve and (B) a sac-like cavity with a blind end containing a giant fecalith.

**Figure 2.**
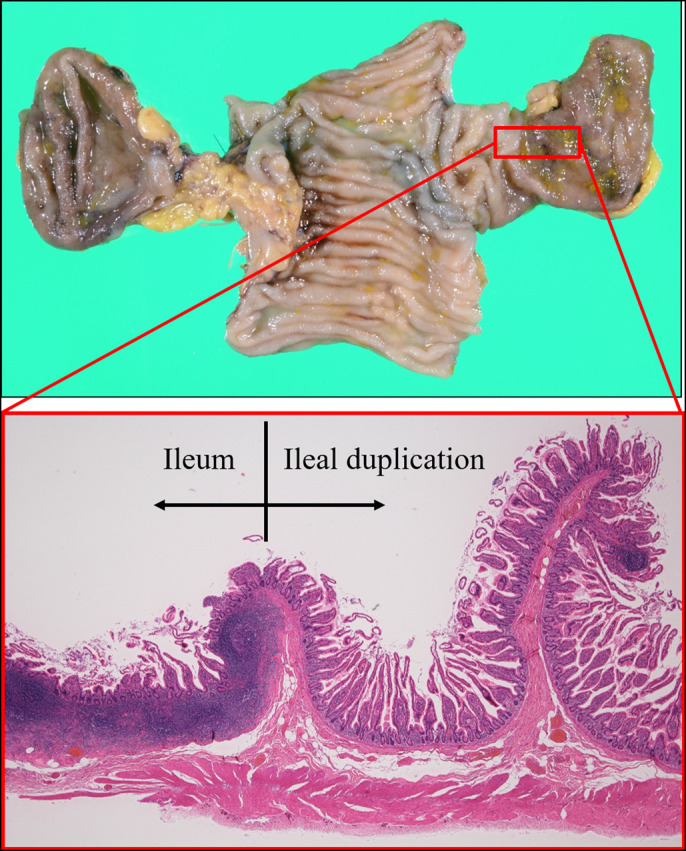
Macroscopic and microscopic observation of the excised specimen showing that the lumen of the sac-like structure has normal small intestinal mucosa, without any ectopic gastric mucosa, pancreatic tissue, erosion, or ulcer.

A 28-year-old man was admitted to our hospital for assessment of the cause of an abnormal calcification shadow that was incidentally detected on an abdominal x-ray image obtained during the treatment of orthopedic back pain. Abdominal computed tomography revealed a sac-like structure on the mesenteric side of the ileum, containing a calcification inside. Retrograde DBE revealed a bifurcated lumen in the ileum, located approximately 100 cm from the ileocecal valve. The narrow lumen had a sac-like cavity with a blind end and contained a hard white-yellowish spherical substance (Figure 1). Small bowel follow-through showed that the sac-like structure was present on the mesenteric side. The patient underwent partial resection of the ileum, including the sac-like structure. The sac-like structure was approximately 5 cm in diameter and had a giant fecalith inside. On microscopic observation, the lumen of the sac-like structure had normal small intestinal mucosa, without any ectopic gastric mucosa, pancreatic tissue, erosion, or ulcer (Figure 2).

Based on these findings, we diagnosed the patient with duplication of the ileum. Here, the unique findings of retrograde DBE aided in making the diagnosis. The present case may be the first case in which ileal duplication with a giant fecalith was directly observed endoscopically. Previously, there have been only 3 reported cases in which intestinal duplication was detected using DBE.^2–4^ All these cases were adults with ileal duplication. Similar to our case, bifurcated lumen with a blind end was detected by retrograde DBE in 2 of these 3 cases.^3,4^ A case reported by Ogino et al only had a diverticular-like luminal opening, and a duplication cyst was confirmed postoperatively.^2^ Our case and previous reports indicate that DBE can be a useful tool for diagnosing intestinal duplication in adults, characterized by either a bifurcated or diverticular-like lumen with a blind end.

## DISCLOSURES

Author contributions: All authors contributed equally to this manuscript. K. Endo is the article guarantor.

Financial disclosure: None to report.

Informed consent was obtained for this case report.
